# Dataset on ammonia, nitrous oxide, methane, and carbon dioxide fluxes from two soils fertilized amended with treated and non-treated cattle slurry

**DOI:** 10.1016/j.dib.2018.10.124

**Published:** 2018-10-27

**Authors:** David Fangueiro, José L.S. Pereira, Irene Fraga, Sónia Surgy, Ernesto Vasconcelos, João Coutinho

**Affiliations:** a LEAF, Instituto Superior de Agronomia, Universidade de Lisboa, Tapada da Ajuda, 1349-017 Lisboa, Portugal; b Escola Superior Agrária de Viseu, Instituto Politécnico de Viseu, Quinta da Alagoa, 3500-606 Viseu, Portugal; c CITAB, Universidade de Trás-os-Montes e Alto Douro, Quinta de Prados, 5000-801 Vila Real, Portugal; d Centro de Química, Universidade de Trás-os-Montes e Alto Douro, Quinta de Prados, 5000-801 Vila Real, Portugal

## Abstract

The current data article presents a set of fluxes of ammonia (NH_3_), nitrous oxide (N_2_O), methane (CH_4_), and carbon dioxide (CO_2_) measured from two different soils under a Mediterranean double-cropping system (oat in autumn/winter followed by maize in spring/summer). The two soils were fertilized using four different treatments: (i) Injection of raw cattle slurry (100 mm depth), (ii) application of raw cattle slurry followed by soil incorporation (20 mm depth), (iii) band application of acidified (pH=5.5) cattle slurry followed by soil incorporation (20 mm depth), and (iv) band application of acidified (pH=5.5) cattle slurry without soil incorporation. A non-amended soil was also considered as control treatment. The data presented here were obtained over a three years experiment between 2012 and 2015. Fluxes were measured in a period between slurry applications to soil (before plant seeding) till crop harvest. The data presented here are supporting the research article “Band application of acidified slurry as an alternative to slurry injection in a Mediterranean double-cropping system: Agronomic effect and gaseous emissions” (Fangueiro et al., 2018).

**Specifications table**

**Table:** 

Subject area	*Agricultural science*
More specific subject area	*Ammonia and greenhouse gases emissions*
Type of data	*Figure.*
How data was acquired	*Dynamic chamber technique with acid trap followed by ammonium quantification for NH*_*3*_*fluxes. Static chamber method followed by quantification of N*_*2*_*O, CH*_*4*_*and CO*_*2*_*fluxes by gas chromatography.*
Data format	*Analyzed as described in. Fangueiro et al.*[1], [4], [6], [7]
Experimental factors	*The sandy soil was a Haplic Arenosol and the sandy loam soil was a Haplic Cambisol. The raw cattle slurry was obtained from the slurry storage pit of a commercial dairy farm. Raw slurry acidification was performed by addition of concentrated sulphuric acid* (pH=5.5). The rates of slurries applied in the assigned treatments were ca. 90 kg N ha^-1^ in autumn (oat crop) and ca. 170 kg N ha^−1^ in spring (maize crop).
Experimental features	*A double cropping system, oat in autumn-winter followed by maize in spring-summer, was established in two different soils (sandy and sandy loam soil). Five treatments were established in each soil:*
	1. *Non-amended soil (Control);* 2. *Injection of raw cattle slurry (100 mm depth) (IS);* 3. *Band application of raw cattle slurry followed by soil incorporation (20 mm depth) (SS);* 4. *Band application of acidified (pH=5.5) cattle slurry followed by soil incorporation (20 mm depth) (AS);* 5. *Band application of acidified (pH=5.5) cattle slurry without soil incorporation (ASS).*
	*Gas fluxes measurements were performed from slurry application to soil till plant harvest.*
Data source location	*Lisboa, Portugal (latitude: 38.708089°, longitude: -9.185001°).*
Data accessibility	*Data are with this article.*
Related research article	*Fangueiro et al.*[1].

**Value of the data**

• There is no, or very limited, data of NH_3_ and greenhouse gas emissions from agricultural soils in Portugal. Hence, this set of data will be useful to establish a first baseline.
• Slurry (animal manure) acidification is performed exclusively in North Europe. The data presented here should be useful for comparison with data obtained in North Europe.
• The data presented here will be useful for stakeholders from Mediterranean countries in order to promote slurry acidification, one of the treatments tested in this experiment.

## 1 Data

**Fig. 1:**
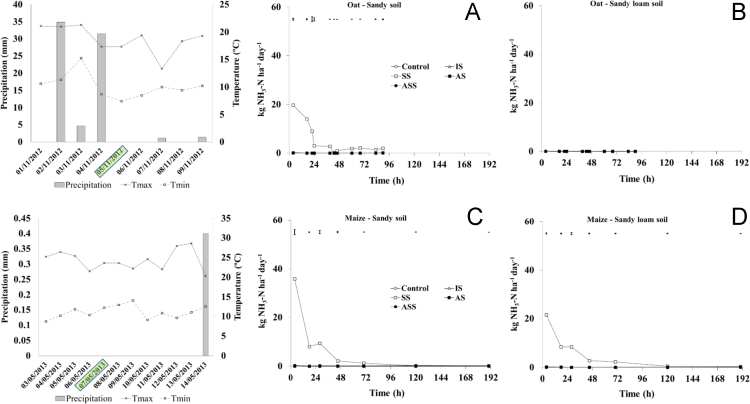
Ammonia daily fluxes following the application of each treatment and meteorological data during the year 2012/2013. Error bars represent the standard error values used for comparison in the Tukey test at each crop (*n*=3). The green box indicate the slurry application date.

**Fig. 2:**
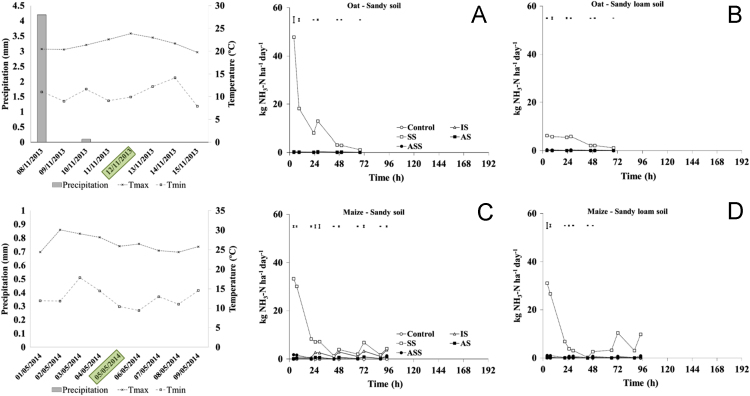
Ammonia daily fluxes following the application of each treatment and meteorological data during the year 2013/2014. Error bars represent the standard error values used for comparison in the Tukey test at each crop (*n*=3). The green box indicate the slurry application date.

**Fig. 3:**
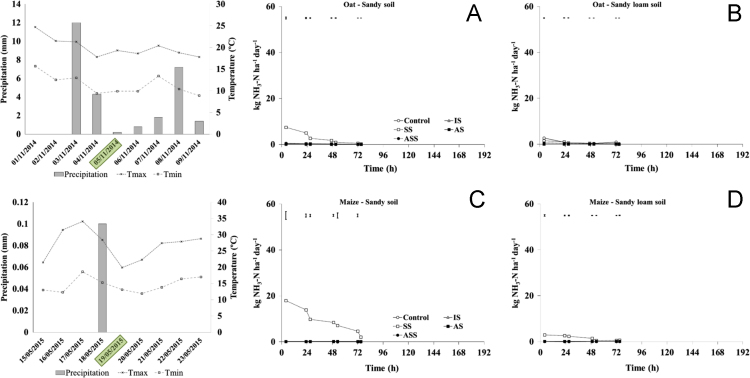
Ammonia daily fluxes following the application of each treatment and meteorological data during the year 2014/2015. Error bars represent the standard error values used for comparison in the Tukey test at each crop (*n*=3). The green box indicate the slurry application date.

**Fig. 4:**
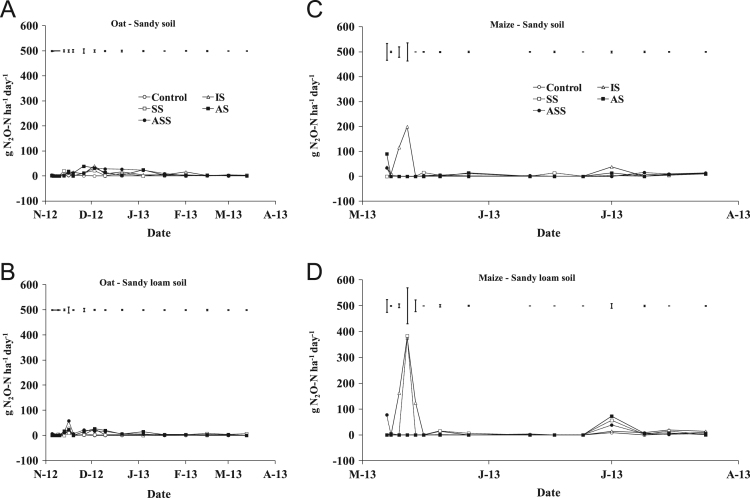
Nitrous oxide daily fluxes following the application of each treatment during the year 2012/2013. Error bars represent the standard error values used for comparison in the Tukey test at each crop (*n*=3).

**Fig. 5:**
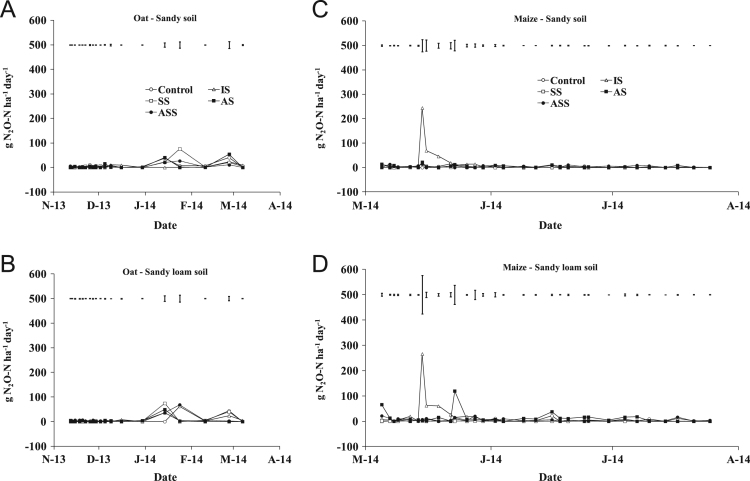
Nitrous oxide daily fluxes following the application of each treatment during the year 2013/2014. Error bars represent the standard error values used for comparison in the Tukey test at each crop (*n*=3).

**Fig. 6:**
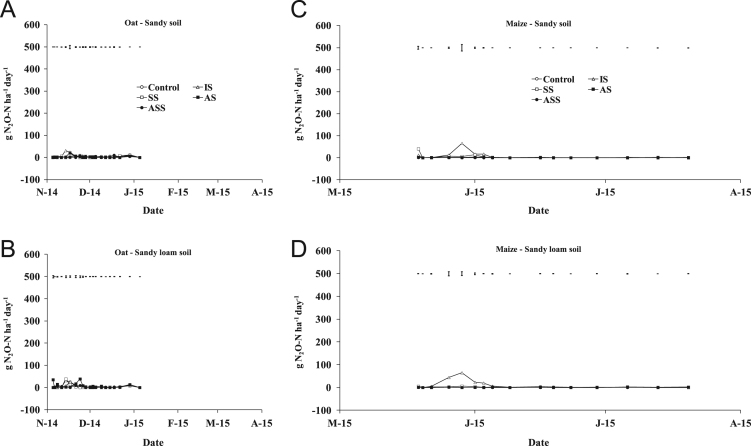
Nitrous oxide daily fluxes following the application of each treatment during the year 2014/2015. Error bars represent the standard error values used for comparison in the Tukey test at each crop (*n*=3).

**Fig. 7:**
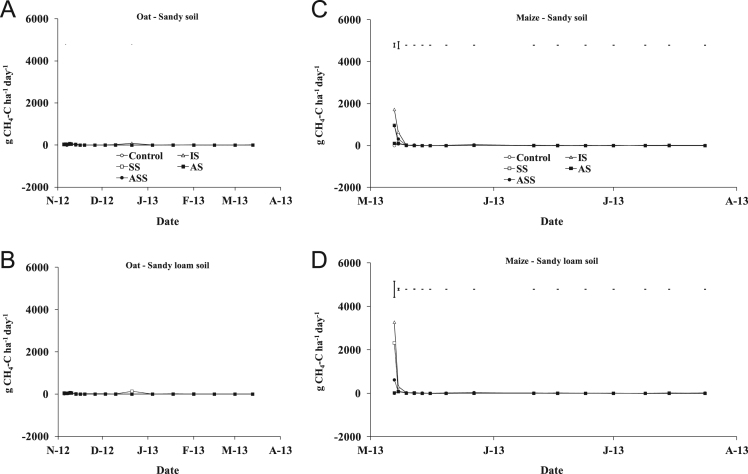
Methane daily fluxes following the application of each treatment during the year 2012/2013. Error bars represent the standard error values used for comparison in the Tukey test at each crop (*n*=3).

**Fig. 8:**
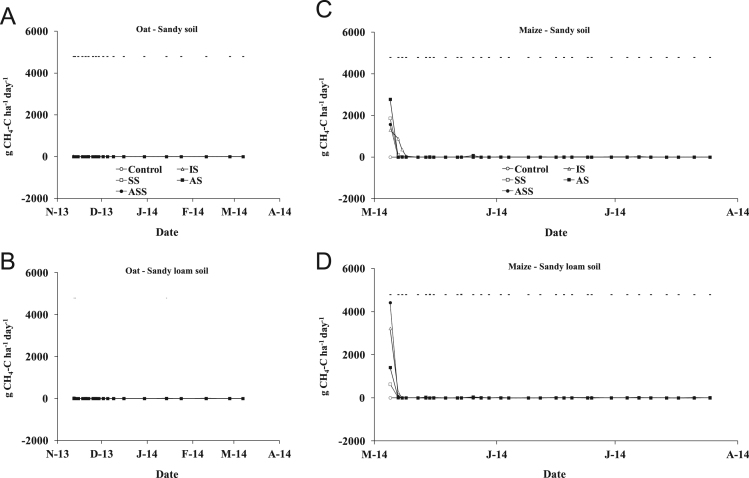
Methane daily fluxes following the application of each treatment during the year 2013/2014. Error bars represent the standard error values used for comparison in the Tukey test at each crop (*n*=3).

**Fig. 9:**
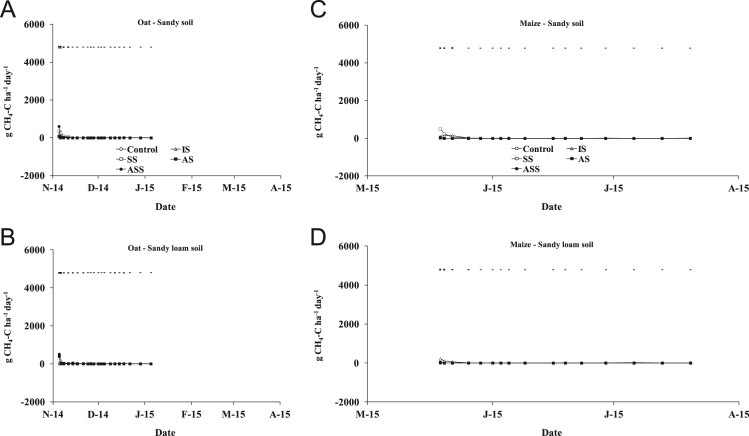
Methane daily fluxes following the application of each treatment during the year 2014/2015. Error bars represent the standard error values used for comparison in the Tukey test at each crop (*n*=3).

**Fig. 10:**
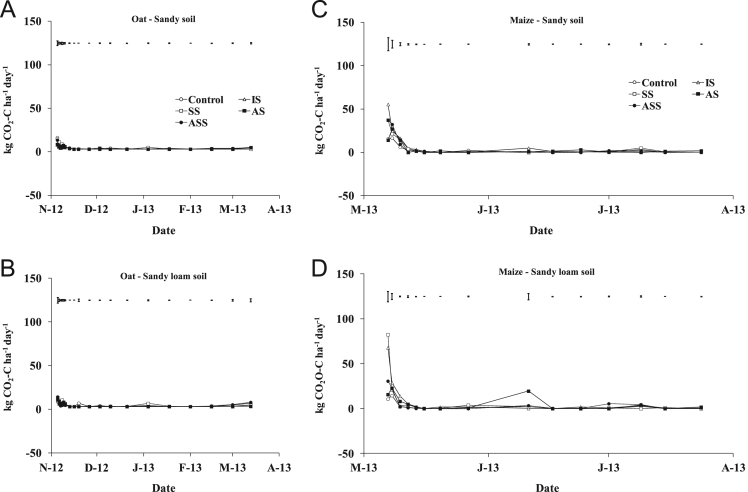
Carbon dioxide daily fluxes following the application of each treatment during the year 2012/2013. Error bars represent the standard error values used for comparison in the Tukey test at each crop (*n*=3).

**Fig. 11:**
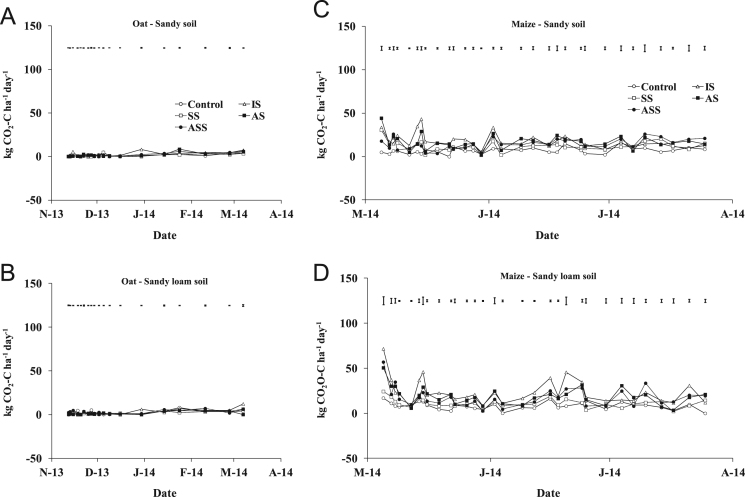
Carbon dioxide daily fluxes following the application of each treatment during the year 2013/2014. Error bars represent the standard error values used for comparison in the Tukey test at each crop (*n*=3).

**Fig. 12:**
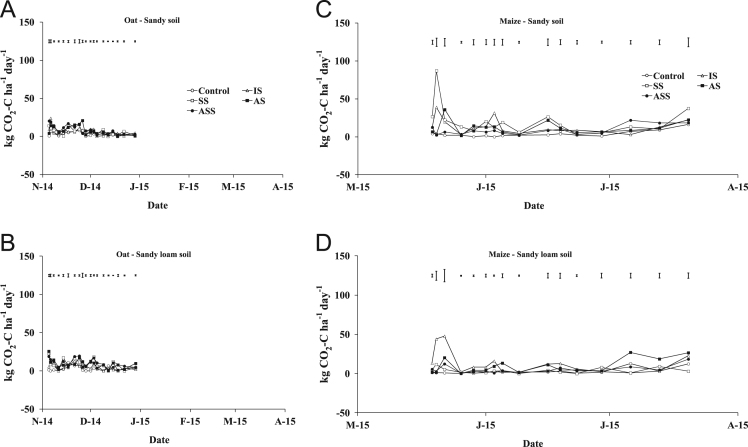
Carbon dioxide daily fluxes following the application of each treatment during the year 2014/2015. Error bars represent the standard error values used for comparison in the Tukey test at each crop (*n*=3).

The present article contains 12 Figures reporting NH_3_, N_2_O, CH_4_, and CO_2_ fluxes measured in two different soils (sandy and sandy-loam soil), during two crops growth (oat: *Avena sativa* L. cv. Saia 6 and maize: *Zea mays* L. FAO 300), and over a three years experiment (2012/2013, 2013/2014, and 2014/2015). Fig. 1, Fig. 2, Fig. 3 present the daily fluxes of NH_3_ following the application of each treatment and meteorological data during the three years of experiment. Fig. 4, Fig. 5, Fig. 6, Fig. 7, Fig. 8, Fig. 9, Fig. 10, Fig. 11, Fig. 12 describe, respectively, the fluxes of N_2_O, CH_4_, and CO_2_ fluxes following the application of each treatment during the three years of experiment.

## 2 Experimental design, materials, and methods

The experiment was carried out at the Instituto Superior de Agronomia (Lisbon, Portugal) (latitude: 38.708089°, longitude: −9.185001°), where a double-cropping system (oat followed by maize) was run over three years (September 2012 to July 2015) in 1 m length × 1 m width × 1 m depth lysimeters filled with two different soils (sandy and sandy-loam soil).

The sandy soil was a Haplic Arenosol [2] with a sandy texture - 700.0 g kg^−1^ coarse sand (0.2–2 mm), 177.0 g kg^−1^ fine sand (0.02–0.2 mm), 97.0 g kg^−1^ silt (0.002–0.02 mm), and 26.0 g kg^−1^ clay (<0.002 mm) - and the main physico-chemical properties of the plough layer (0–300 mm) were: pH (H_2_O): 7.1, organic matter: 5.6 g kg^−1^ dry soil, P_2_O_5_: 40.7 mg kg^−1^ dry soil and K_2_O: 32.3 mg kg^−1^ dry soil. The sandy-loam soil was a Haplic Cambisol [2] with a sandy-loam texture (271.0 g kg^−1^ coarse sand, 558.0 g kg^−1^ fine sand, 72.0 g kg^−^^1^ silt and 99.0 g kg^−1^ clay) and the following principal physico-chemical properties of the plough layer: pH (H_2_O): 6.1, organic matter: 10.7 g kg^−^^1^ dry soil, P_2_O_5_: 32.1 mg kg^−^^1^ dry soil and K_2_O: 114.0 mg kg^−1^ dry soil.

The raw cattle slurry used in this study was obtained from the concrete slurry storage pit of a commercial dairy farm located near Palmela (Portugal) and was kept at ambient temperature in plastic barrels for approximately one week before application. In the 24 h before soil application of the treatments, raw cattle slurry acidification was performed by addition of concentrated sulphuric acid (about 6 mL per L of slurry) to reach a final pH of 5.5, following the procedure described by Fangueiro et al. [3]. The details of the standard analytical methods used to assess the physico-chemical properties of the soils and slurries studied are available in Fangueiro et al. [4].

The rates of slurries applied in the assigned treatments were ca. 90 kg N ha^−1^ in autumn (oat crop) and ca. 170 kg N ha^−1^ in spring (maize crop). The injection of raw slurry was simulated by the manual opening of small grooves (H = 80 mm, L = 300 mm) in the assigned plots, followed by slurry enclosure. The treatments were applied homogenously and/or incorporated by hand in the plots.

The traditional double-cropping forage system, growing oat (*Avena sativa* L. cv. Saia 6) from November to March, followed by hybrid maize (*Zea mays* L. FAO 300) between May and July, was established and both crops were grown according to commercial practice. The seeding rate for both crops was the same in the three consecutive years: 10 plants m^−2^ for maize (750 mm × 115 mm) and 71 plants m^−^^2^ for oat. Maize was irrigated while oat was rain fed only.

The NH_3_ fluxes were measured by the dynamic chamber technique during almost the first 72 h after soil amendment, while the N_2_O, CH_4_ and CO_2_ fluxes were measured by the closed chamber technique during the whole growing period (from cattle slurry application till harvest) [5]. A detailed description of the methods used to assess gas fluxes can be found in Fangueiro et al. [1], [4], [6], [7]. Briefly, the NH_3_ fluxes in each plot were measured using a circular polyvinyl chloride chamber (Ø = 210 mm, H = 55 mm) placed randomly and for measuring the N_2_O, CH_4_, and CO_2_ fluxes, one square polyvinyl chloride chamber (L = 230 mm, H = 240 mm) was inserted into the soil immediately after slurry application [4].

A meteorological station (Delta-T Devices, Cambridge, UK) located in the experimental site was used to collect precipitation and minimum and maximum air temperature data during the experimental period.

Tukey comparisons of means (*p*<0.05) were carried out for the factors “soil” as a split-plot on “treatments” and factor “year” as a split-plot on factor “soil” and their interactions using the statistical software package STATISTIX 7.0 (USA).
